# Effect of Sling Suspension-Based Active Shoulder Joint Exercises on Shoulder Subluxation in Subacute Stroke Patients

**DOI:** 10.7759/cureus.109397

**Published:** 2026-05-21

**Authors:** Mukta T Killedar, Suraj B Kanase

**Affiliations:** 1 Department of Neurosciences, Krishna College of Physiotherapy, Krishna Vishwa Vidyapeeth (Deemed to be University), Karad, IND

**Keywords:** hemiplegic shoulder, motor function recovery, randomized controlled trial, shoulder exercises, shoulder joint stability, shoulder subluxation, sling suspension, stroke physiotherapy, subacute stroke, upper limb recovery

## Abstract

Introduction

Shoulder subluxation has an incidence of up to 80% following stroke. It commonly occurs during the flaccid stage due to reduced muscle tone in the rotator cuff muscles, allowing gravitational pull on the humerus and resulting in widening of the acromion-humeral gap. Sling suspension enables partially unloaded active movement of the shoulder joint; however, evidence regarding its effectiveness in subacute stroke patients remains limited. This study aimed to evaluate the effect of sling suspension-based active shoulder exercises on shoulder subluxation in subacute stroke patients.

Methods

A randomized controlled trial with a pretest-posttest design was conducted on 28 subacute stroke patients who were randomly allocated to experimental and control groups (n = 14 each). Both groups received a standard rehabilitation program consisting of weight-bearing and scapular stabilization exercises for the affected upper limb. In addition, the experimental group performed sling suspension-based active shoulder exercises, in which the limb was supported in a suspension system to facilitate gravity-minimized active movements. The control group performed conventional active-assisted range of motion (AAROM) exercises with therapist assistance, without sling support. All participants received 30 minutes of intervention per session, five days per week for four weeks. Shoulder subluxation was assessed using the measuring tape method (acromion-humeral distance) by a blinded outcome assessor. Data were analyzed using IBM SPSS Statistics version 31.0 (IBM Corp., Armonk, NY). Within-group comparisons were performed using the paired t-test, and between-group comparisons of mean difference were analyzed using the independent samples t-test. A p-value of <0.05 was considered statistically significant.

Results

Both groups demonstrated a significant reduction in shoulder subluxation following the intervention; however, the experimental group showed significantly greater improvement than the control group. The experimental group demonstrated a mean reduction of 6.3 ± 1.2 mm, whereas the control group demonstrated a mean reduction of 2.3 ± 0.9 mm. Between-group analysis revealed a statistically significant difference favoring the experimental group (mean difference: 4.0 mm; p < 0.001).

Conclusion

The findings indicate that sling suspension-based active shoulder exercises, when combined with standard rehabilitation, produce greater reductions in shoulder subluxation than conventional AAROM exercises in individuals with subacute stroke.

## Introduction

Musculoskeletal dysfunction is a well-recognized consequence of stroke, frequently presenting as shoulder subluxation, pain, spasticity, and contracture formation. Among these, shoulder subluxation remains one of the most prevalent complications in individuals with hemiparesis, with incidence rates reported to reach up to 80% [1]. This condition generally arises during the initial flaccid phase, primarily due to hypotonia and the inability of periarticular muscles to provide adequate dynamic stabilization to the glenohumeral joint. In the absence of sufficient motor recovery, the condition may persist or progress further during later stages characterized by spasticity and increased muscle stiffness [2]. Clinically, it is identified as an inferior displacement of the humeral head relative to the glenoid fossa, which is commonly quantified using the acromion-humeral distance [3]. This measurement serves as a standardized and objective indicator for assessing the severity of subluxation and monitoring therapeutic outcomes [4]. Furthermore, deficits in trunk control during the flaccid stage often lead to asymmetrical posture, with a tendency to shift toward the affected side, thereby increasing mechanical loading on the shoulder joint and contributing to balance disturbances in some patients with hemiplegia [5].

The clinical significance of shoulder subluxation extends beyond structural displacement, as it plays a major role in the development of secondary musculoskeletal and neurovascular complications. Disruption of normal joint alignment alters shoulder biomechanics, resulting in excessive strain on surrounding soft tissues, including ligaments, muscles, the joint capsule, neural elements, and vascular structures. These alterations can manifest as pain, restricted joint mobility, and delayed functional recovery [6,7]. In addition, abnormal positioning of the humeral head increases the likelihood of associated conditions such as impingement, tendinopathy, adhesive capsulitis, and possible involvement of the brachial plexus [8,9]. Collectively, these impairments limit effective use of the affected upper limb during daily activities, ultimately reducing functional independence and negatively influencing rehabilitation outcomes. Therefore, early identification and appropriate management of shoulder subluxation are critical components of post-stroke rehabilitation.

A variety of conservative management strategies have been employed to address shoulder subluxation, including supportive slings, neuromuscular electrical stimulation, therapeutic taping, and positioning devices such as arm supports and wheelchair attachments [10-13]. Slings are widely utilized to provide external support, maintain joint alignment, and enhance proprioceptive awareness of limb positioning [12]. However, interventions that rely solely on passive support may not adequately address the underlying neuromuscular deficits associated with stroke. In contrast, sling suspension systems promote active engagement by reducing the effects of gravity and minimizing friction, thereby facilitating controlled and graded movement of the affected limb [14].

Sling exercise therapy represents an active rehabilitation approach that integrates muscle strengthening, range of motion training, neuromuscular re-education, and sensorimotor integration [15]. These systems allow adjustment of support levels and enable movement across multiple planes, thereby encouraging progressive muscle activation in individuals with impaired motor control [14]. Previous research has demonstrated that sling-based interventions can improve balance and enhance upper limb performance in stroke populations. However, most available studies have predominantly focused on trunk stability or lower limb function, with relatively limited emphasis on their role in managing shoulder subluxation specifically [14,16,17].

Shoulder subluxation is also closely associated with deficits in voluntary motor control of the upper limb, underscoring the need for targeted therapeutic interventions [18,19]. Evidence from both clinical observations and imaging studies suggests that sling-based exercise programs can improve glenohumeral alignment, highlighting their therapeutic potential [16,17]. More recent findings indicate that active shoulder exercises performed using sling suspension systems may contribute to a reduction in subluxation and improvements in upper limb function in individuals during the subacute phase of stroke recovery [18]. Additionally, comparative studies have reported favorable outcomes associated with sling-supported training in enhancing upper limb recovery among patients with hemiparesis [19,20].

Despite these promising results, a considerable portion of the existing literature has compared sling-based interventions with technologically advanced rehabilitation approaches such as robotic-assisted therapy. Although effective, such methods are often costly and may not be readily accessible in many clinical settings. Consequently, there is a need to evaluate sling suspension-based exercises in comparison with simpler, widely available conventional treatment approaches to determine their true clinical applicability and effectiveness.

The present study addresses these gaps by focusing on practical and accessible rehabilitation strategies. Firstly, it compares sling suspension-based active shoulder exercises with active-assisted range of motion (AAROM) exercises, both of which are commonly used and cost-effective in routine physiotherapy practice. The study specifically included individuals in the subacute phase of stroke (seven days to six months post-stroke). Although participants within this phase may demonstrate varying degrees of spontaneous neurological recovery, randomized group allocation and standardized rehabilitation protocols were used to minimize potential recovery-related bias while evaluating the effects of the intervention. Although substantial research has been conducted in the acute and chronic phases, the subacute stage remains relatively underexplored, particularly with respect to targeted upper limb rehabilitation.

Accordingly, the objective of this study is to evaluate the effectiveness of sling suspension-based active shoulder joint exercises in reducing shoulder subluxation in individuals with subacute stroke and to compare these outcomes with those achieved through AAROM exercises. It was hypothesized that sling suspension-based active shoulder joint exercises would result in a greater reduction in shoulder subluxation compared to AAROM exercises. The findings are expected to contribute to the development of practical, cost-effective, and evidence-based rehabilitation strategies aimed at optimizing upper limb recovery during a crucial phase of post-stroke rehabilitation.

## Materials and methods

This investigation was conducted as a randomized, parallel-group experimental trial from November 5, 2025, to March 31, 2026, at the Krishna College of Physiotherapy, Neurosciences Outpatient Department (OPD), Karad. The purpose of the study was to determine the effectiveness of sling suspension-based active shoulder joint exercises in reducing shoulder subluxation among individuals in the subacute stage of stroke. Approval for the study was obtained from the Institutional Ethics Committee on November 5, 2025, prior to initiation. A randomized controlled framework was adopted to enhance internal validity, establish a causal relationship between intervention and outcome, and minimize potential selection bias.

Participants were recruited using a consecutive sampling approach from patients attending the Neurosciences OPD. The sample size of 28 participants was determined based on feasibility, availability of eligible participants during the study period, and institutional time constraints. A formal a priori power analysis was not performed due to the feasibility-based nature of the study and the limited availability of eligible participants. Randomization was carried out using sealed opaque envelopes to ensure proper allocation concealment. Eligible individuals were provided with detailed information regarding the study objectives and procedures, and written informed consent was obtained before inclusion.

The study population consisted of patients in the subacute stage of stroke, defined as the period ranging from seven days to six months following stroke onset, presenting with shoulder subluxation, operationally defined as an acromion-humeral distance exceeding 11 mm between the acromion and the humeral head [21].

Inclusion criteria comprised participants between 50 and 65 years of age who were hemodynamically stable, able to follow simple verbal commands, possessed adequate sitting balance to perform the intervention protocol, and demonstrated sufficient motor function to actively participate in shoulder exercises. Participants were additionally required to have no prior orthopedic disorders affecting the shoulder complex.

Exclusion criteria included shoulder pain in the absence of subluxation, pre-existing shoulder pathology, fractures or traumatic injuries of the affected shoulder, and severe motor impairment below fair grade (muscle strength graded as poor or less).

A total of 28 participants were randomly assigned to two groups: an experimental group receiving sling suspension-based active shoulder exercises and a control group receiving AAROM exercises. The intervention was carried out over four weeks, with sessions conducted five times per week.

Statistical analysis was performed using IBM SPSS Statistics version 31.0 (IBM Corp., Armonk, NY). Descriptive statistics were used to summarize the data. Continuous variables, including age and shoulder subluxation (acromion-humeral distance), were expressed as mean and standard deviation. Categorical variables, including gender and side affected, were presented as frequencies and percentages.

The normality of data distribution was assessed using the Shapiro-Wilk test. The paired t-test was applied to evaluate within-group differences between pre- and post-intervention values. The independent t-test was used to compare continuous variables between the experimental and control groups. The chi-square test was used to analyze categorical variables. A p-value of <0.05 was considered statistically significant.

**Figure 1:**
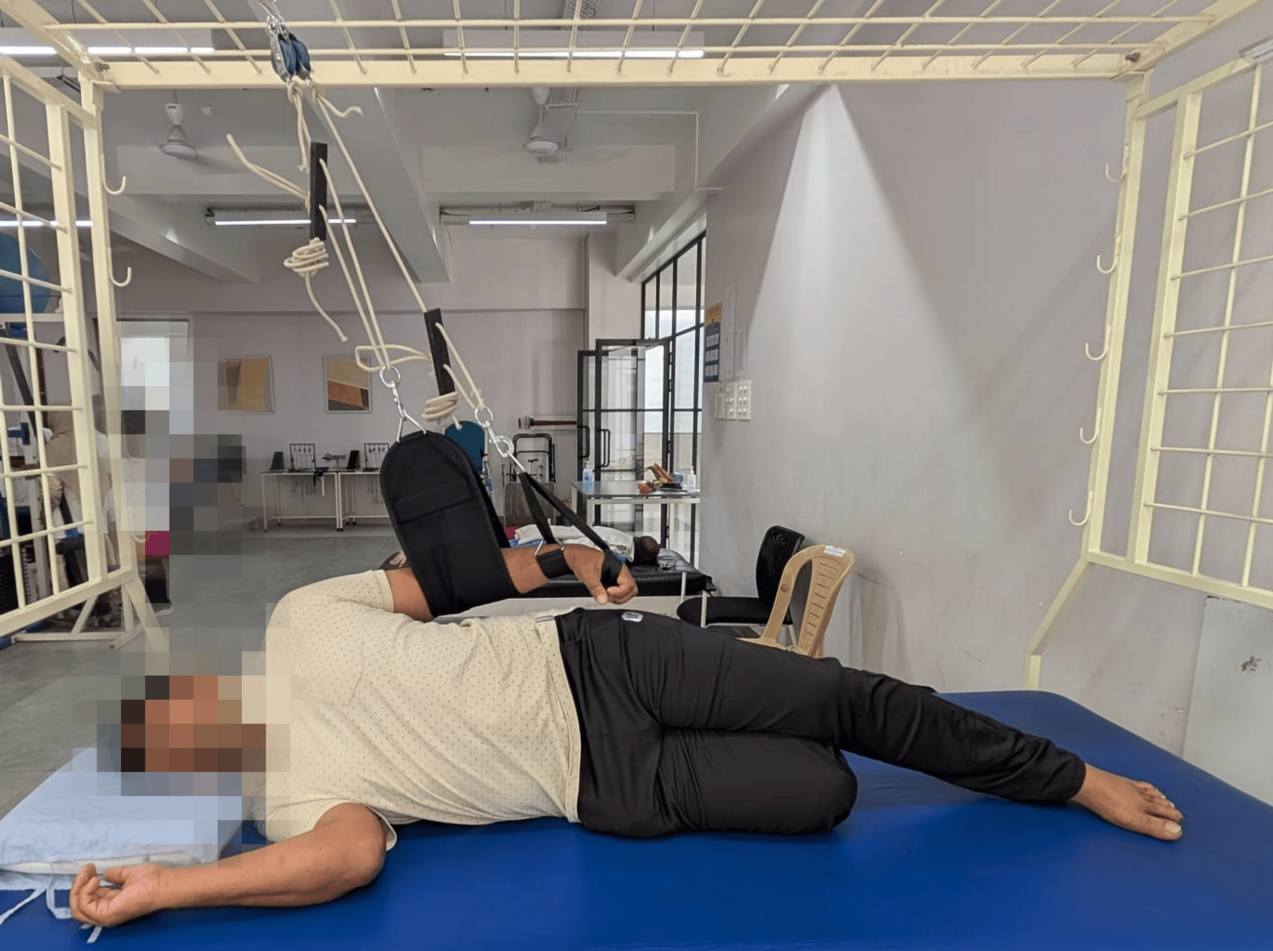
Sling suspension-based active shoulder flexion and extension exercises performed in side-lying position with partial unloading of the affected upper limb.

The sling suspension-based exercise program included a series of gravity-minimized, active shoulder movements performed in different positions. The side-lying sling suspension-based active shoulder flexion-extension exercise was performed with the patient positioned in side-lying, allowing controlled flexion and extension movements (Figure 1).

**Figure 2:**
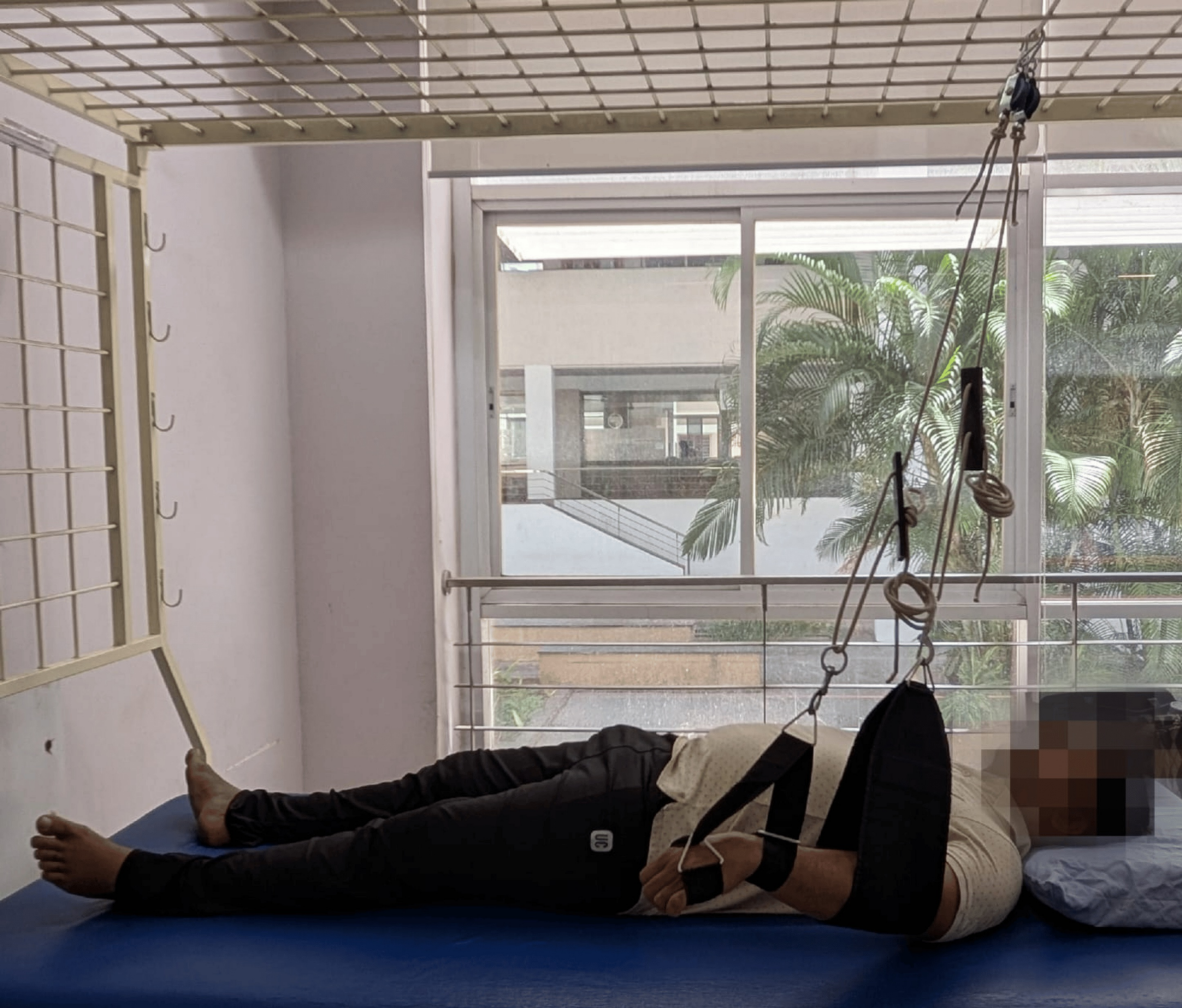
Sling suspension-based active shoulder abduction and adduction exercises performed in the supine position with partial unloading of the affected upper limb.

The supine sling suspension-based active shoulder abduction-adduction exercise enabled active abduction and adduction movements in a gravity-minimized plane (Figure 2).

**Figure 3:**
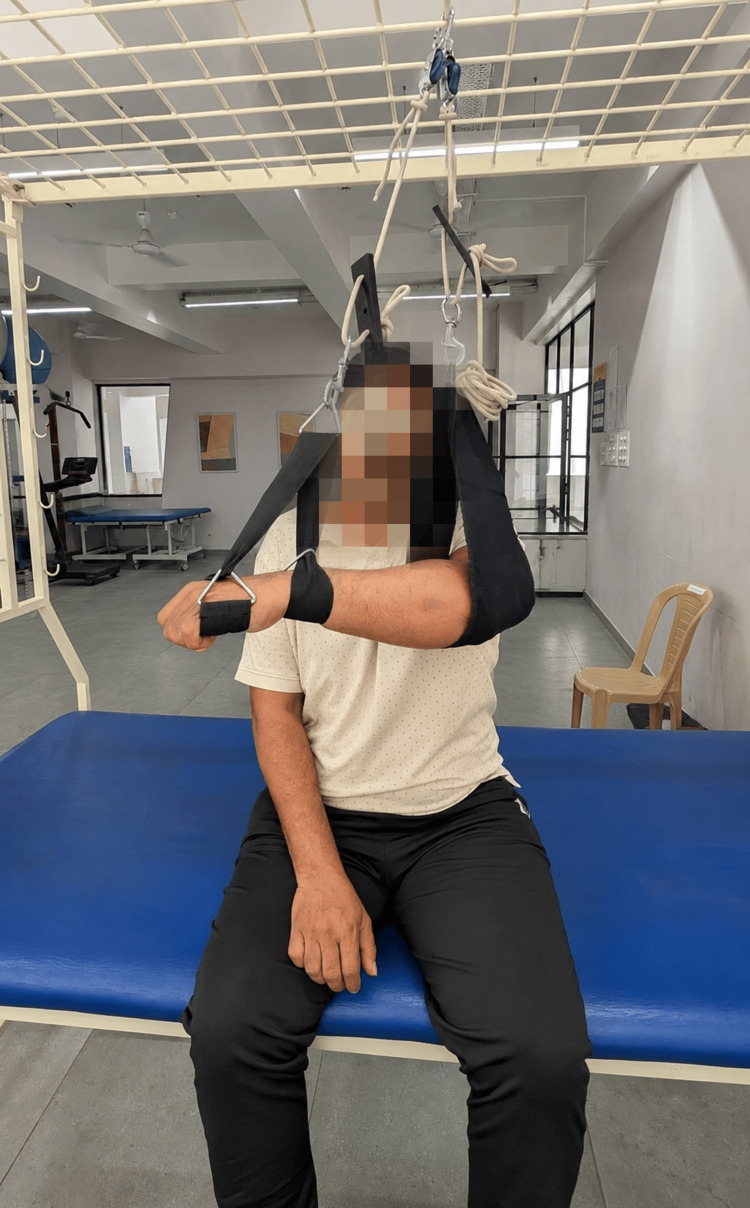
Sling suspension-based active horizontal shoulder adduction exercise in sitting position with partial unloading of the affected upper limb.

For horizontal adduction and abduction, the sling suspension system was adjusted to provide partial unloading of the affected upper limb, thereby reducing gravitational load while still permitting active muscle recruitment. The patient was positioned in sitting with the shoulder at approximately 90° of flexion, facilitating active horizontal adduction (Figure 3).

**Figure 4:**
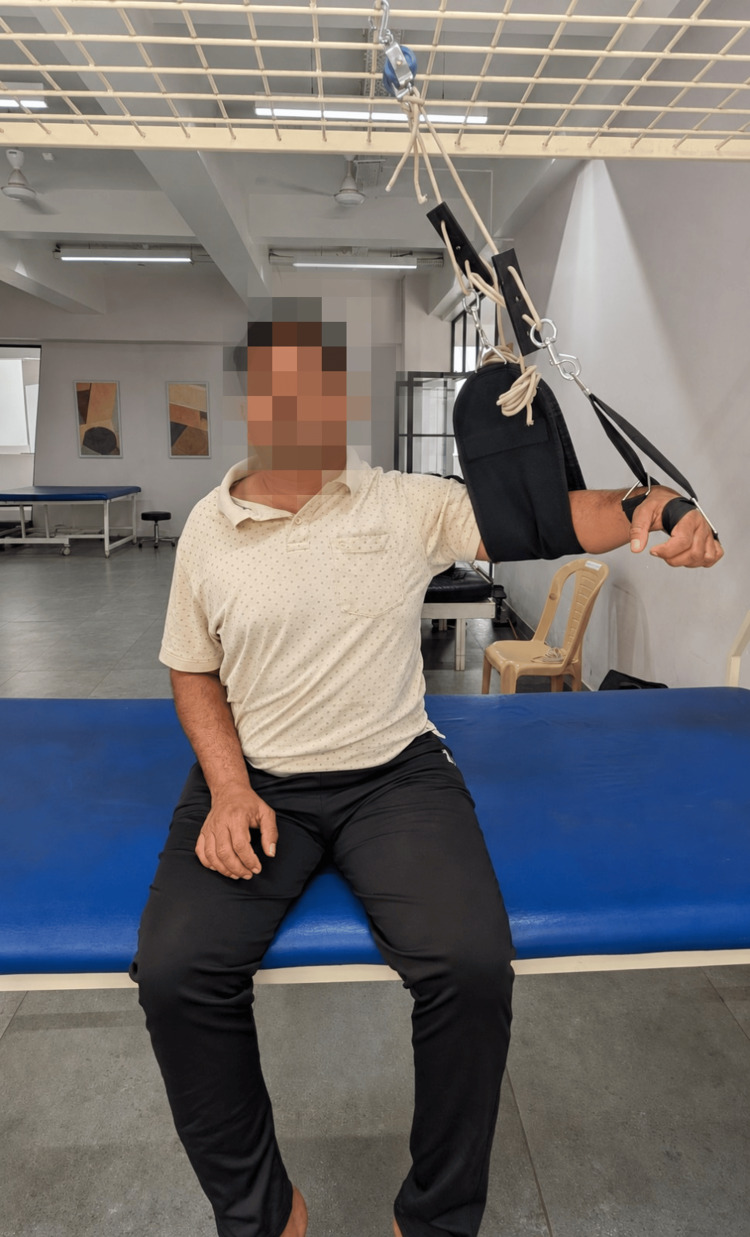
Sling suspension-based active horizontal shoulder abduction exercise in sitting position with partial unloading of the affected upper limb.

For horizontal abduction, the patient was positioned in sitting with the shoulder at approximately 90° of flexion, facilitating active horizontal abduction (Figure 4).

**Figure 5:**
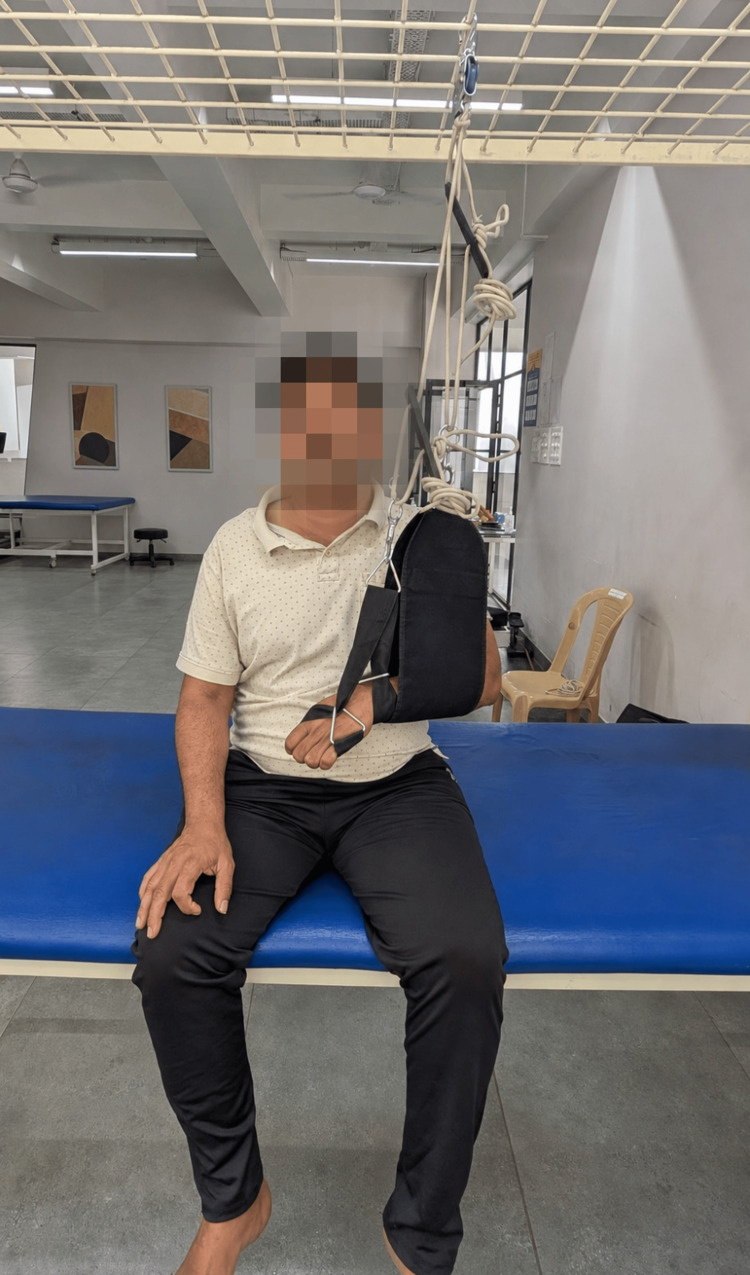
Sling suspension-based active shoulder internal rotation exercise performed in sitting position with partial unloading of the affected upper limb.

Rotational movements were also performed in sitting with the shoulder in a neutral position and the elbow flexed. Active internal rotation of the shoulder was performed in a sitting position with the shoulder maintained in neutral and the elbow flexed (Figure 5). 

**Figure 6:**
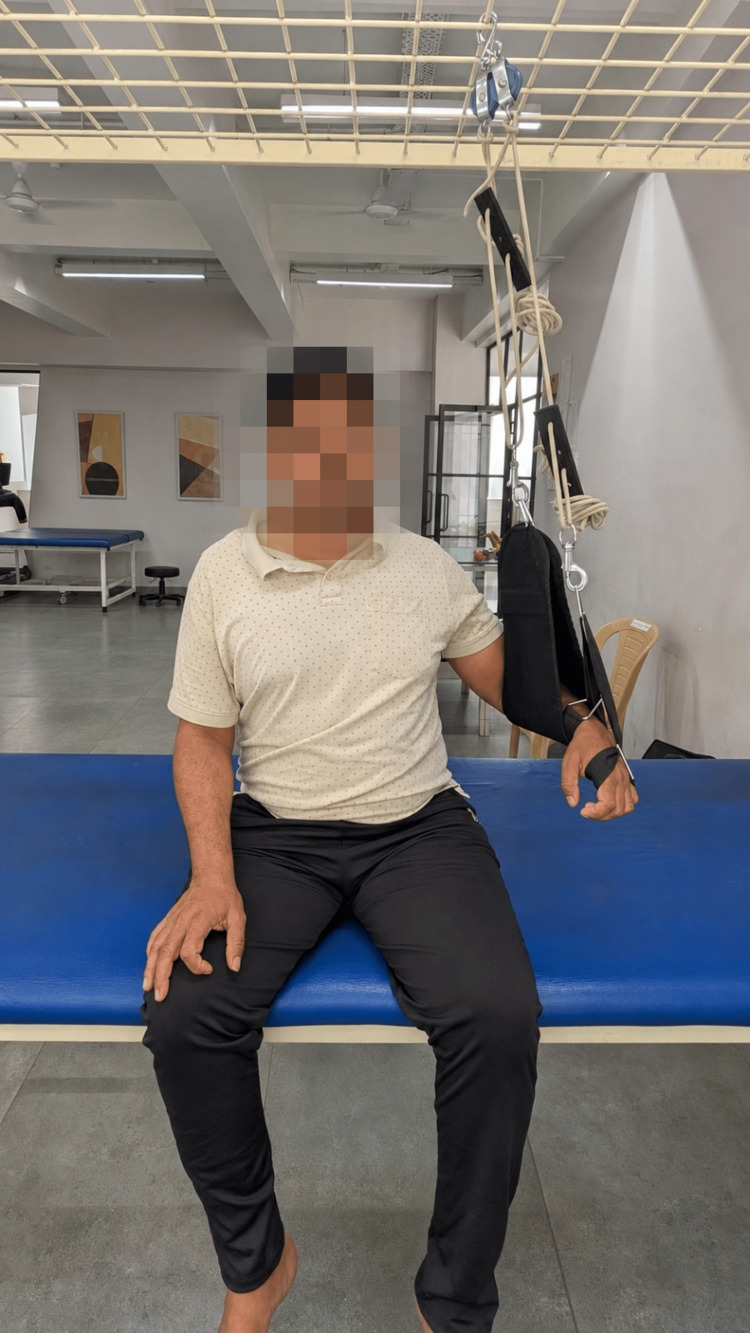
Sling suspension-based active shoulder external rotation exercise performed in sitting position with partial unloading of the affected upper limb.

Active external rotation of the shoulder was performed in a sitting position with the shoulder maintained in neutral and the elbow flexed (Figure 6). 

The sling suspension system was utilized due to its ability to partially support the upper limb, decrease gravitational loading, and enable controlled, multidirectional active movements, thereby facilitating selective muscle activation and improved joint positioning. The control intervention, consisting of AAROM exercises, was selected as it reflects a standard, cost-effective therapeutic approach commonly applied in clinical rehabilitation settings. The detailed structure of the exercise protocol, including positioning, duration, frequency, and progression, is presented in Table 1.

**Table 1:** Comparison of standard rehabilitation and group-specific shoulder exercise interventions in experimental (sling suspension-based active shoulder exercises) and control (AAROM exercises) groups. AAROM: active-assisted range of motion

Exercise/Type	Control Group	Experimental Group
Active-Assisted Range of Motion (AAROM)	The patient is positioned in a supine lying position on a plinth with the head supported and the affected upper limb relaxed. All AAROM exercises are performed by the therapist in a pain-free range, ensuring proper scapulohumeral rhythm and avoiding compensatory trunk movements. The therapist stands on the affected side and provides manual assistance through appropriate hand placement at the distal humerus/elbow and wrist/forearm, depending on the movement pattern.	Not included
	Shoulder Movements Performed:	
	· Flexion-Extension: The therapist assists the patient in moving the shoulder from neutral into available flexion range (up to tolerance) and returns it to extension in a controlled manner.	
	· Abduction-Adduction: The limb is guided in the frontal plane away from and back toward the body while maintaining alignment and preventing shoulder hiking or trunk compensation.	
	· Internal-External Rotation: With the shoulder in neutral and elbow flexed to 90°, the forearm is moved outward (external rotation) and inward (internal rotation) while the therapist stabilizes the elbow to isolate glenohumeral movement.	
	Each movement is performed for 20 repetitions per set.	
	Progression:	
	· Assistance is gradually reduced from maximal to minimal support.	
	· Patient participation is progressively increased	
	· Available range of motion is increased as tolerated	
	· All movements are maintained within a pain-free range	
Sling Suspension-Based Active Shoulder Exercises	Not included	All exercises are performed using a sling suspension system, in which the affected upper limb is supported through straps attached to the distal forearm (just proximal to the wrist joint) and hand region (metacarpal level). In selected cases, an additional strap may be used around the mid-forearm to improve stability and alignment. The sling system is adjusted to ensure that the entire upper limb is partially unloaded against gravity, while maintaining the shoulder in a functional, pain-free alignment that allows free movement in all planes without traction stress at the glenohumeral joint.
		The sling height is set such that the limb remains aligned with the trunk, preventing shoulder elevation or downward pull. This configuration facilitates controlled active movement with reduced gravitational resistance.
		Exercises are performed in a pain-free range, with emphasis on scapulohumeral rhythm, smooth motor control, and prevention of compensatory trunk movements. The therapist stands on the affected side of the patient throughout the intervention and continuously monitors scapular position, joint alignment, and movement quality. Manual facilitation is provided at the scapula (proximal control) and forearm/wrist region (distal control) when required.
		Shoulder Movements Performed:
		· Shoulder Flexion-Extension (Side-Lying Position)
		The patient lies in a side-lying position on the non-affected side, with the affected upper limb supported in the sling at the distal forearm and hand region, allowing unrestricted sagittal plane movement. The therapist stands at shoulder level on the affected side, stabilizing the scapula and guiding the forearm when required. The patient actively performs shoulder flexion by moving the arm forward and upward, followed by controlled extension backward. The sling reduces gravitational load and facilitates smooth movement initiation.
		Repetitions: 20
		· Shoulder Abduction-Adduction (Supine Position)
		The patient lies in supine position, with the affected upper limb supported in the sling at the distal forearm and hand region, positioned close to the body at rest. The sling is adjusted to allow free frontal plane movement without shoulder stress. The therapist stabilizes the scapula and assists distal control if needed. The patient actively moves the arm away from the midline into abduction and returns it to adduction in a controlled manner.
		Repetitions: 20
		· Shoulder Internal-External Rotation (Sitting Position)
		The patient sits upright with the affected upper limb supported in the sling at the distal forearm and hand region, with the elbow maintained at 90° flexion and shoulder in neutral position. The therapist monitors from the affected side and stabilizes the elbow or forearm when required. The patient performs external rotation by moving the forearm outward and internal rotation by bringing it toward the abdomen, enabling isolated glenohumeral movement.
		Repetitions: 20
		· Shoulder Horizontal Abduction-Adduction (Sitting Position)
		The patient sits upright with trunk in neutral alignment, and the affected upper limb supported in the sling at the distal forearm and hand region, adjusted at shoulder height. The therapist stands slightly behind the affected side to monitor scapular control and trunk stability. The patient actively moves the arm horizontally away from the midline and returns it to the starting position in a controlled manner.
		Repetitions: 20
		Progression:
		·Assistance is gradually reduced from maximal to minimal support
		· Patient participation is progressively increased
		· Available range of motion is increased as tolerated
		· All movements are maintained within a pain-free range
Weight-Bearing Exercises	Quadruped Position (Anterior-Posterior and Lateral Weight Shifts)	Quadruped Position (Anterior-Posterior and Lateral Weight Shifts)
	· Patient position	· Patient position
	The patient assumes a quadruped (hands-and-knees) position with the affected upper limb in full elbow extension, wrist in neutral, and palm placed firmly on the support surface. Proper alignment is maintained through the wrist, elbow, and shoulder, with scapula in neutral position.	The patient assumes a quadruped (hands-and-knees) position with the affected upper limb in full elbow extension, wrist in neutral, and palm placed firmly on the support surface. Proper alignment is maintained through the wrist, elbow, and shoulder, with scapula in neutral position.
	· Therapist position	· Therapist position
	The therapist stands on the affected side of the patient to continuously monitor scapular stability, trunk alignment, and overall posture.	The therapist stands on the affected side of the patient to continuously monitor scapular stability, trunk alignment, and overall posture.
	· Handling	· Handling
	Manual support is provided at the scapula and/or pelvis when required to prevent shoulder collapse, scapular winging, or trunk asymmetry. Verbal and tactile cues are used to maintain proper weight distribution.	Manual support is provided at the scapula and/or pelvis when required to prevent shoulder collapse, scapular winging, or trunk asymmetry. Verbal and tactile cues are used to maintain proper weight distribution.
	· Exercise performance	· Exercise performance
	The patient performs slow, controlled anterior-posterior and lateral weight shifts within a pain-free range while maintaining stability and alignment.	The patient performs slow, controlled anterior-posterior and lateral weight shifts within a pain-free range while maintaining stability and alignment.
	Repetitions: 20	Repetitions: 20
	Bedside Sitting Position (Weight Bearing on Affected Upper Limb)	Bedside Sitting Position (Weight Bearing on Affected Upper Limb)
	· Patient position	· Patient position
	The patient sits upright at the edge of the plinth with feet supported on the floor. The affected upper limb is placed on the plinth beside the hip with elbow extended, wrist in neutral, and palm in contact with the surface, allowing graded weight bearing.	The patient sits upright at the edge of the plinth with feet supported on the floor. The affected upper limb is placed on the plinth beside the hip with elbow extended, wrist in neutral, and palm in contact with the surface, allowing graded weight bearing.
	· Therapist position	· Therapist position
	The therapist stands on the affected side to ensure optimal trunk alignment and scapular control throughout the activity.	The therapist stands on the affected side to ensure optimal trunk alignment and scapular control throughout the activity.
	· Handling	· Handling
	Manual contact is provided at the scapula and trunk to prevent lateral trunk lean, shoulder hiking, or loss of midline orientation. Assistance is adjusted according to patient stability.	Manual contact is provided at the scapula and trunk to prevent lateral trunk lean, shoulder hiking, or loss of midline orientation. Assistance is adjusted according to patient stability.
	· Exercise performance	· Exercise performance
	The patient performs controlled weight shifts onto the affected upper limb while maintaining upright posture and scapular stability.	The patient performs controlled weight shifts onto the affected upper limb while maintaining upright posture and scapular stability.
	Repetitions: 20	Repetitions: 20
Scapular Stabilization Exercises	Shoulder Shrugs (Elevation-Depression)	Shoulder Shrugs (Elevation-Depression)
	· Patient position	· Patient position
	The patient was positioned in an upright sitting posture on a plinth with feet supported on the floor and trunk maintained in neutral alignment. The affected upper limb was relaxed alongside the body.	The patient was positioned in an upright sitting posture on a plinth with feet supported on the floor and trunk maintained in neutral alignment. The affected upper limb was relaxed alongside the body.
	· Therapist position	· Therapist position
	The therapist stood on the affected side to monitor scapular movement and postural alignment.	The therapist stood on the affected side to monitor scapular movement and postural alignment.
	· Handling / Facilitation	· Handling / Facilitation
	· Exercise performance	· Exercise performance
	The patient actively performed shoulder elevation by shrugging both shoulders upward followed by controlled relaxation into the neutral position in a slow and controlled manner within a pain-free range.	The patient actively performed shoulder elevation by shrugging both shoulders upward followed by controlled relaxation into the neutral position in a slow and controlled manner within a pain-free range.
	Repetitions: 20	Repetitions: 20
	Shoulder Protraction and Retraction	Shoulder Protraction and Retraction
	· Patient position	· Patient position
	The patient was seated upright with trunk in neutral alignment and shoulders relaxed.	The patient was seated upright with trunk in neutral alignment and shoulders relaxed.
	· Therapist position	· Therapist position
	The therapist stood on or slightly behind the affected side to observe scapular mechanics and ensure correct movement patterns.	The therapist stood on or slightly behind the affected side to observe scapular mechanics and ensure correct movement patterns.
	· Handling	· Handling
	Manual guidance was provided along the medial and lateral borders of the scapula when required to facilitate proper protraction and retraction while minimizing trunk compensation.	Manual guidance was provided along the medial and lateral borders of the scapula when required to facilitate proper protraction and retraction while minimizing trunk compensation.
	· Exercise performance	· Exercise performance
	The patient actively performed scapular protraction by moving the shoulders forward and retraction by drawing them backward in a controlled and smooth manner within a pain-free range.	The patient actively performed scapular protraction by moving the shoulders forward and retraction by drawing them backward in a controlled and smooth manner within a pain-free range.
	Repetitions: 20	Repetitions: 20

The primary outcome variable was the degree of shoulder subluxation, quantified by measuring the distance between the acromion and the humeral head in millimeters using a standardized clinical method. This measurement technique was chosen for its practicality, affordability, and sensitivity in detecting changes in acromion-humeral alignment. Shoulder subluxation was assessed clinically using this method, and radiographic (X-ray) confirmation was not utilized in this study. Assessments were performed at two time points: prior to the initiation of treatment (baseline) and following completion of the four-week intervention period (post-intervention). To reduce the risk of measurement bias, all evaluations were conducted by a blinded outcome assessor. 

## Results

A comparison of pre- and post-intervention shoulder subluxation demonstrated that both groups showed improvement; however, the experimental group exhibited a significantly greater reduction compared to the control group. The experimental group (Group A) showed a mean reduction of 6.3 ± 1.2 mm, which was statistically significant (t = 8.42, p < 0.001), indicating a very large effect. In contrast, the control group (Group B) demonstrated a smaller but statistically significant improvement of 2.3 ± 0.9 mm (t = 3.25, p = 0.004), representing a moderate effect. Furthermore, between-group analysis revealed a statistically significant difference (t = 5.12, p < 0.001) with a mean difference of 4.0 mm, favoring the experimental group, as shown in Table 2.

**Table 2:** Comparison of within-group and between-group changes in shoulder subluxation following intervention (n = 14 per group). Within-group comparisons (pre- vs post-intervention) were analyzed using the paired t-test. Between-group comparison of mean difference in shoulder subluxation was performed using the independent samples t-test. Data are presented as mean ± standard deviation(mm). A p-value of <0.05 was considered statistically significant. mm: millimeter; SD: standard deviation

Group	Pre-test Mean ± SD (mm)	Post-test Mean ± SD (mm)	Mean Difference ± SD (mm)	t-value	p-value	Cohen’s d Value	Effect Size (Interpretation)
Experimental	18.4 ± 2.1	12.1 ± 1.8	6.3 ± 1.2	8.42	<0.001	5.25	Very large improvement
Control	17.9 ± 2.4	15.6 ± 2.1	2.3 ± 0.9	3.25	0.004	2.56	Moderate improvement
Between-group comparison of mean difference (change from pre-test to post-test)	—	—	4	5.12	<0.001	4	Greater improvement favoring the experimental group

The gender distribution of participants was comparable between the experimental and control groups, with no statistically significant difference (χ² = 0.14, p = 0.71), indicating homogeneity of the sample, as presented in Table 3.

**Table 3:** Distribution of gender among participants in experimental and control groups. Chi-square test was used to compare the distribution of gender between the experimental and control groups; p < 0.05 was considered statistically significant. n: number of participants

Gender	Experimental (n = 14)	Control (n = 14)	χ² value	p-value
Male	8	7	0.14	0.71
Female	6	7	—	—

The mean age of participants in both groups was comparable, with no statistically significant difference (t = 0.36, p = 0.72), indicating baseline equivalence between groups, as shown in Table 4.

**Table 4:** Comparison of age distribution between experimental and control groups. An independent samples t-test was used to compare the mean age between the experimental and control groups; p < 0.05 was considered statistically significant. n: number of participants; SD: standard deviation

Group	Mean Age (Years)	SD	t-value	p-value
Experimental (n = 14)	58.3	6.2	0.36	0.72
Control (n = 14)	58.9	6	—	—

The distribution of the affected side (right/left) was similar between the two groups, with no statistically significant difference (χ² = 0.14, p = 0.71), confirming baseline homogeneity, as illustrated in Table 5.

**Table 5:** Comparison of side affected (right/left) between experimental and control groups. Chi-square test was used to compare the distribution of the affected side between the experimental and control groups; p < 0.05 was considered statistically significant. n: number of participants

Side Affected	Experimental (n = 14)	Control (n = 14)	χ² value	p-value
Right	7	8	0.14	0.71
Left	7	6	—	—

Baseline shoulder subluxation values were comparable between the experimental and control groups, with no statistically significant difference (t = 0.59, p = 0.56), confirming equivalence prior to intervention, as presented in Table 6.

**Table 6:** Baseline comparison of shoulder subluxation (acromion-humeral distance) between groups. An independent samples t-test was used to compare baseline shoulder subluxation distance between the experimental and control groups; p < 0.05 was considered statistically significant. mm: millimeter; SD: standard deviation

Group	Baseline Shoulder Subluxation Mean (mm)	SD	t-value	p-value
Experimental	18.4	2.1	0.59	0.56
Control	17.9	2.4	—	—

## Discussion

This study utilized a randomized controlled design to assess the effectiveness of sling suspension-based active shoulder exercises in reducing shoulder subluxation in individuals with subacute stroke. The findings demonstrated a significantly greater reduction in acromion-humeral distance in the experimental group compared to the AAROM group. Specifically, the experimental group showed a mean reduction of 6.3 mm, while the control group improved by 2.3 mm. Between-group comparison of mean difference revealed a statistically significant difference of 4.0 mm (t = 5.12, p < 0.001), favoring the experimental group. These results indicate that active movement with partial unloading of the upper limb using sling suspension is more effective in restoring glenohumeral alignment than assisted movement alone. As secondary outcomes were not included, the conclusions are limited to changes in shoulder alignment rather than functional recovery.

The present results align with existing literature on sling-based rehabilitation. Jung and Choi [17] reported notable improvements in shoulder alignment and upper limb function following sling suspension exercises in individuals with acute stroke. Similarly, Kim et al. [18] demonstrated the beneficial effects of sling-based rehabilitation in improving upper limb motor performance and shoulder stability in stroke patients. More recent evidence by Kim et al. [22] further supports the effectiveness of elastic dynamic sling interventions in reducing shoulder subluxation and improving upper limb function in individuals with subacute stroke. In contrast to these studies, which primarily evaluated advanced or technology-assisted rehabilitation approaches, the current study compared sling suspension exercises with AAROM, two simple, low-cost interventions frequently used in clinical practice. This comparison enhances the practical applicability of the findings, particularly in rehabilitation settings with limited access to specialized equipment.

The improvements observed in the experimental group may be explained by several underlying mechanisms. The sling suspension system was adjusted to provide partial unloading of the affected upper limb, reducing gravitational demand while allowing voluntary active movement. This environment facilitates improved positioning of the humeral head within the glenoid cavity and minimizes compensatory movement patterns. Furthermore, active engagement of the rotator cuff and shoulder stabilizing muscles during sling-assisted exercises likely contributes to improved joint stability and neuromuscular coordination. Enhanced proprioceptive input during active movement may also support better sensorimotor integration. In contrast, AAROM primarily involves therapist-assisted movement, which may limit voluntary muscle activation and reduce proprioceptive feedback, potentially explaining the comparatively smaller improvements observed.

Recent evidence on gravity-modulated and unloading-based rehabilitation approaches has further emphasized the importance of reducing gravitational load to facilitate upper limb motor recovery. Celian et al. [23] investigated wearable anti-gravity support systems for upper limb rehabilitation in stroke populations and reported effects on upper limb movement performance, although functional recovery outcomes remained variable. These findings support the biomechanical rationale underlying the present intervention, where partial unloading through sling suspension allowed safer and more controlled active movement.

Focusing on the subacute phase of stroke recovery (seven days to six months) adds important clinical value to this study. Much of the previous literature has emphasized the acute stage of stroke recovery, during which rapid spontaneous neurological recovery may obscure the true effects of rehabilitation interventions. By examining patients in the subacute phase - when spontaneous recovery begins to plateau but neuroplasticity remains active - the present study provides a clearer understanding of the direct effects of sling suspension-based exercise. This stage is clinically important for preventing secondary musculoskeletal complications and promoting optimal upper limb recovery.

Within the broader evidence base, Ada and Foongchomcheay [11] demonstrated that electrical stimulation can effectively reduce shoulder subluxation following stroke. The magnitude of improvement observed in the present study appears comparable, suggesting that sling suspension-based active exercises may offer similar therapeutic benefits without the need for specialized electrotherapeutic equipment. However, comparisons across studies should be interpreted cautiously because of variations in intervention protocols, outcome measures, and participant characteristics.

This study has several strengths, including a randomized controlled design, allocation concealment using sealed opaque envelopes, and blinded outcome assessment, which enhance internal validity. The inclusion of a practical control group further strengthens clinical relevance. However, several limitations must be acknowledged. The intervention period was relatively short, limiting long-term interpretation. Functional outcomes such as motor recovery and activities of daily living were not assessed, and therefore, the findings are restricted to changes in shoulder alignment. Future studies should incorporate functional outcome measures such as the Fugl-Meyer Assessment (FMA) to determine whether improvements in shoulder alignment correlate with upper limb motor recovery and functional performance. Furthermore, shoulder subluxation was clinically measured using acromion-humeral distance, and radiographic (X-ray) confirmation was not performed, which may limit measurement precision. The sample size was small and based on feasibility without a priori power analysis, which may affect generalizability.

From a clinical standpoint, the results support the inclusion of sling suspension-based active exercises in rehabilitation programs for subacute stroke patients. This approach is cost-effective, non-invasive, and practical for implementation in standard physiotherapy settings. Incorporating such active interventions during this critical recovery phase may help improve shoulder alignment, enhance neuromuscular control, and reduce the risk of secondary complications, thereby contributing to more effective rehabilitation outcomes.

## Conclusions

Sling suspension-based active shoulder joint exercises are significantly more effective than conventional AAROM exercises in reducing shoulder subluxation in subacute stroke patients. The greater reduction in acromion-humeral distance suggests improved glenohumeral alignment and enhanced joint stability. Given its cost-effectiveness, non-invasive nature, and feasibility in routine clinical settings, this intervention may be considered a valuable addition to stroke rehabilitation programs. However, as functional outcome measures were not assessed in the present study, future research with larger sample sizes and the incorporation of functional assessments such as the FMA is recommended to establish long-term effectiveness and broader clinical applicability.
